# Effect of New-Onset Diabetes Mellitus on Renal Outcomes and Mortality in Patients with Chronic Kidney Disease

**DOI:** 10.3390/jcm7120550

**Published:** 2018-12-14

**Authors:** Po-Ke Hsu, Chew-Teng Kor, Yao-Peng Hsieh

**Affiliations:** 1 Department of Internal Medicine, Changhua Christian Hospital, Changhua 50006, Taiwan; 180358@cch.org.tw (P.-K.H.); 179297@cch.org.tw (C.-T.K.); 2 School of Medicine, Kaohsiung Medical University, Kaohsiung 80708, Taiwan; 3 School of Medicine, Chung Shan Medical University, Taichung 40201, Taiwan

**Keywords:** chronic kidney disease (CKD), end-stage renal disease (ESRD), incident diabetes, mortality, new-onset diabetes mellitus (DM)

## Abstract

Background: The incidence rates of diabetes mellitus (DM) and chronic kidney disease (CKD) are increasing worldwide and their coexistence can have a large negative impact on clinical outcomes. However, it is unclear how incident DM affects CKD patients. Methods: Incident CKD patients between 2000 and 2013 were identified from the National Health Insurance Research Database of Taiwan; they were classified as non-DM (*n* = 10,356), pre-existing DM (*n* = 6982), and incident DM (*n* = 1103). Non-DM cases were patients who did not develop DM before the end of the observation period. The outcomes of interest were end-stage renal disease (ESRD), mortality, and composite outcome (ESRD or death). The association between the DM groups and clinical outcomes was estimated using the inverse probability of group-weighted (IPW) multivariate-adjusted time-dependent Cox regression models. Results: During the study period of 14 years, 1735 (16.6%) patients in the non-DM group reached ESRD compared with 2168 (31.05%) in the pre-existing DM group and 111 (11.03%) in the incident DM group (*p* < 0.001). Moreover, 2219 (21.43%) patients in the non-DM group died compared with 1895 (27.14%) in the pre-existing DM group and 303 (27.47%) in the incident DM group (*p* < 0.001). Compared with the non-DM group, the pre-existing DM group was associated with a higher risk of ESRD [hazard ratio (HR) 2.54; 95% confidence interval (CI 2.43–2.65), death (HR 2.23; 95% CI 2.14–2.33), and a composite outcome (HR 2.29; 95% CI 2.21–2.36). Similarly, incident DM was also associated with a higher risk of ESRD (HR 1.12; 95% CI 1.06–1.19), death (HR 2.48; 95% CI 2.37–2.60), and a composite outcome (HR 1.77; 95% CI 1.70–1.84) compared with the non-DM group. Factors contributing to incident DM included old age, low monthly income, and having hypertension, hyperlipidemia, and ischemic heart disease, while pentoxifylline reduced the risk of incident DM. Conclusion: Similarly to pre-existing DM, CKD patients with incident DM carried a higher risk of ESRD, mortality, and a composite outcome compared with those with non-DM. For those at risk of incident DM, strict monitoring and intervention strategies must be adopted to help improve their clinical outcomes.

## 1. Introduction

Chronic kidney disease (CKD) is an important global health issue due to the increased recognition of its progression to end-stage renal disease (ESRD), its high cardiovascular burden, and high mortality rates [1]. Patients with CKD are more likely to die, mainly from cardiovascular disease (CVD), than to progress to ESRD; the risk of death is actually more than two times higher than the risk of reaching ESRD among patients with advanced stage 4 CKD [2]. A reduced estimated glomerular filtration rate (eGFR) and increased albuminuria have been identified as significant risk factors for all-causes and CVD death in the general population and CKD patients [3,4,5,6]. Their co-existence confers to a multiplicative risk of mortality. The strong association between CKD and an adverse CVD prognosis prompted the National Kidney Foundation and the American College of Cardiology/American Heart Association to recommend CKD as a risk equivalent to coronary heart disease (CHD) [7,8].

Similarly to CKD, the world prevalence of diabetes is estimated to exceed 430 million adults globally by 2030 [9]. The mortality rate of diabetic patients is more than twice that of patients without diabetes mellitus (DM) [10]. Many studies have identified DM as a strong predictor of CVD mortality, leading the clinical guidelines to designate DM with a risk equivalent to CHD and place it in the riskiest category [11]. In addition, DM is also a risk factor for the development of CKD and accounts for the majority of CKD cases [12]. Diabetic nephropathy (DN) accounts for 40% of ESRD patients. Compared with non-diabetic patients, diabetic patients have a higher risk of ESRD, and this trend persists even if ESRD is attributed to causes other than diabetes [13]. Therefore, concurrent DM and CKD lead to an extremely high risk of CVD morbidity, mortality, and ESRD [14].

It has long been known that CKD disrupts the homeostasis of glucose and insulin regulation; Boer et al. reported that non-diabetic CKD patients had a reduced insulin sensitivity and insufficient compensatory insulin secretion, which led to impaired glucose tolerance in 65% of subjects [15]. Therefore, DM causes CKD and vice versa. However, how incident DM affects renal outcomes and patient survival among CKD patients has not yet been identified. Therefore, the present study was conducted to retrospectively compare the risk of ESRD and mortality between CKD patients with pre-existing DM, non-DM, and incident DM using nationwide population data.

## 2. Materials and Methods

### 2.1. Data Source

The Taiwan National Healthcare Insurance (NHI) Program has existed since 1995 and covers >99% of Taiwanese residents. The National Health Insurance Research Database (NHIRD) of Taiwan is derived from the reimbursement claims within the NHI program and contains all the information on the medical practices performed. The Longitudinal Health Insurance Database (LHID) was randomly sampled from the NHIRD and includes all the longitudinally linked data of the 1 million enrollees. A nationwide retrospective population-based cohort study was conducted using the LHID 2005 of Taiwan. The International Classification of Diseases, Ninth Revision, Clinical Modification (ICD-9-CM) is used for the reimbursement of medical expenses. Therefore, the information contained within the NHIRD regarding medical care has been well validated, and a variety of high-standard articles have been previously published using data from the NHIRD of Taiwan [16,17]. The present study was carried out in compliance with the declaration of Helsinki and was approved by the Institutional Review Board of Changhua Christian Hospital. A retrospective study conducted in Taiwan does not require the informed consent of the participants.

### 2.2. Study Cohort and Design

The present study was conducted to investigate how the DM status of CKD patients affected their clinical outcomes. Comorbidities were considered present if there were at least two appropriate ICD-9-CM medical codes within one year at outpatient visits and the interval between the first and last date of medical coding was at least 90 days apart or there was one diagnostic code in the hospitalization dataset. The diagnosis of DM (ICD-9 code 250) also required the prescription of glucose-lowering agents, including either insulin or oral glucose-lowering drugs. First, 25,130 patients with CKD between 1996 and 2013 were identified. Then, those diagnosed with CKD before 2000 were excluded to leave the incident CKD patients from 2000 to 2013. The ICD9-CM codes used to define CKD, DM, and comorbidities are presented in Supplementary Table S1. Patients with CKD who were <18 years old, >100 years old, had dialysis-dependent ESRD, had received a renal transplant, had incomplete demographic data, or <90 days of follow-up were also excluded from the study. Patients were followed from the first date of CKD diagnosis to the date of death or the end of the study on 31 December 2013. New-onset DM was defined as DM diagnosed after the index date of CKD diagnosis, whereas pre-existing DM was DM diagnosed before the onset of CKD. The remaining CKD patients were designated as the non-DM group.

### 2.3. Outcome Measures and Relevant Variables

The primary outcomes of interest were ESRD requiring renal replacement therapy and mortality, while the secondary outcome was a composite of ESRD or mortality. The causes of death for CKD patients were also looked at according to their DM status. CVD and infection were the pre-specific causes of death for analysis. The main discharge diagnosis was considered the cause of death if a patient died during the admission and the first discharge diagnosis of the last hospitalization within three months before death was assigned to the cause of death outside the hospital. Covariates that were known to be important determinants for the study outcomes were retrieved for statistical analysis; this included medical conditions diagnosed within one year before study enrollment, drug treatments, demographic data, and the frequency of outpatient visits.

### 2.4. Statistical Analyses

The distribution of the patient’s baseline characteristics in the study population was presented as a number (proportion) for categorical data and as the mean ± standard deviation or median and interquartile range for continuous data. The differences between the three DM groups were compared using one-way analysis of variance (ANOVA), the Chi-squared test, or Fisher’s exact test, as appropriate. Observed event rates were recorded by DM groups and the cumulative incidence of event rates for each group was expressed for every 1000 patient-years. The time at risk of the event started when incident CKD was first diagnosed. For the identification of incident DM, CKD patients must survive long enough to develop DM. The so-called immortal time bias occurs because they are immortal by definition before exposure. The time-dependent Cox regression model was used to circumvent this bias, and the risk of death was also considered a competing event while analyzing the risk of ESRD.

While considering the confounding caused by the different distribution of observed covariates, a generalized boosted regression model was used to calculate the propensity scores for the DM groups based on all the baseline characteristics, which yielded the optimal covariate balance between the three groups. Next, the inverse probability of group-weighted (IPW) study populations was estimated using the calculated propensity scores from generalized boosted regression [18,19]. The balance of covariate distribution was evaluated using standardized differences. If a standardized difference of <10% was reached after IPW, the covariates were considered balanced. The DM status was not a fixed categorical covariate, as it changed from non-DM status to DM status for the incident DM group during the observation period. Therefore, the Simon and Makuch method was used, an alternative to the Kaplan–Meier estimate, to compute the graphical survival curves for the three DM groups.

The associations between DM groups and study outcomes were expressed with hazard ratios (HRs) and 95% confidence intervals (CIs) using the IPW-standardized time-dependent cause-specific Cox model that accounted for immortal time bias, the competing risk of death, and covariate balance. Significant variables associated with new-onset DM in non-pre-existing DM patients with CKD were determined. All statistical analyses were performed using R language with SPSS statistical software, version 20.0 (IBM Corp., Armonk, NY, USA) and SAS 9.4 software (SAS Institute Inc., Cary, NC, USA). All tests were two-tailed and a *p*-value < 0.05 was considered to indicate a statistically significant difference.

## 3. Results

### 3.1. Patient Characteristics

**Figure 1:**
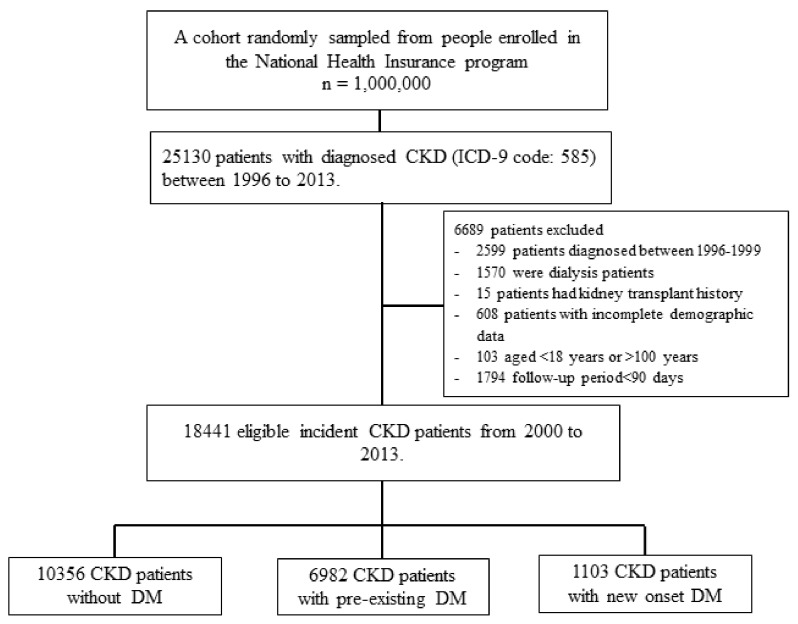
Flowchart of patient selection processes for incident chronic kidney disease (CKD) with non-DM, pre-existing DM and incident DM. DM = diabetes mellitus.

Patients with CKD between 2000 and 2013 were enrolled in the study, as depicted in Figure 1. Overall, a total of 18,441 incident CKD patients met the inclusion criteria and were categorized by their DM status into the non-DM (*n* = 10,356), pre-existing DM (*n* = 6982), or incident DM (*n* = 1103) groups. The follow-up time was 4.96 ± 3.89, 3.88 ± 3.10, and 4.20 ± 3.54 years for the non-DM, pre-existing DM, and incident DM, respectively (*p* < 0.001). Prior to the inverse probability of group weighting, there were statistically significant differences among the DM groups across most baseline covariates, see Table 1. In general, the pre-existing DM group had the highest proportion of cardiovascular comorbidities, followed by the incident DM group, and then the non-DM group. After IPW, all the covariates were well balanced between the DM groups, with the maximum between group-standardized differences <10%.

### 3.2. Long-Term Risk of ESRD by DM Status

**Figure 2:**
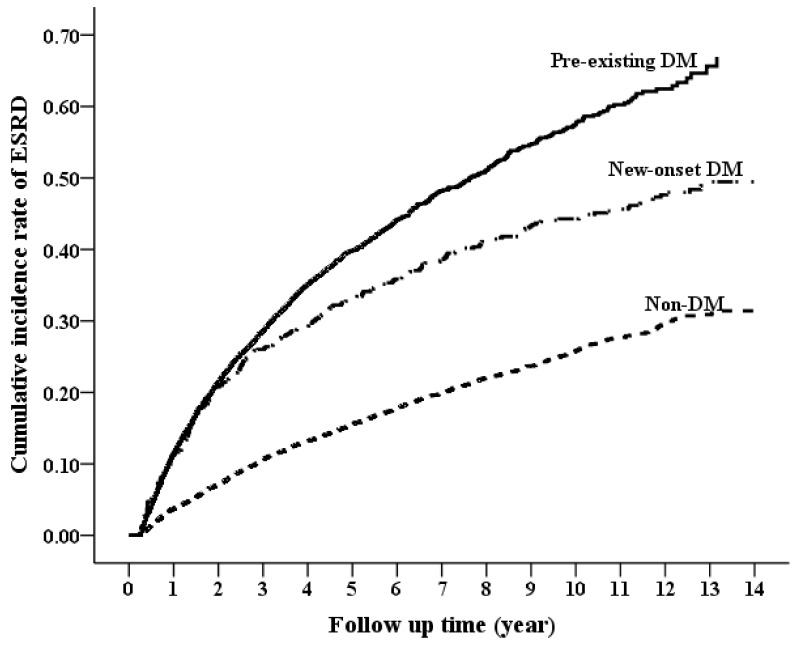
Cumulative incidence rate of progression to end-stage renal disease between pre-existing DM, non-DM, and incident DM groups.

During the study period of 14 years, 1735 (16.6%) patients in the non-DM group reached ESRD compared with 2168 (31.05%) in the pre-existing group and 111 (11.03%) in the incident group (*p* < 0.001). The cumulative ESRD probability was plotted for the DM groups using the Simon and Makuch method, see Figure 2. The incidence rate of ESRD was 101.28 per 1000 person-years for the pre-existing DM group, 34.49 per 1000 person-years for the incident DM group, and 33.36 per 1000 person-years for the non-DM group, see Table 2. Pre-existing DM was associated with a 154% higher risk of ESRD (HR 2.54; 95% CI 2.43–2.65), whereas incident DM was associated with a 12% higher risk of ESRD (HR 1.12; 95% CI 1.06–1.19) when compared with the non-DM group. When compared with the incident DM group, pre-existing DM was associated with a 130% higher risk of ESRD development (HR 2.30; 95% CI 2.17–2.44; Supplementary Table S2).

### 3.3. Long-Term Risk of Mortality by DM Status

**Figure 3:**
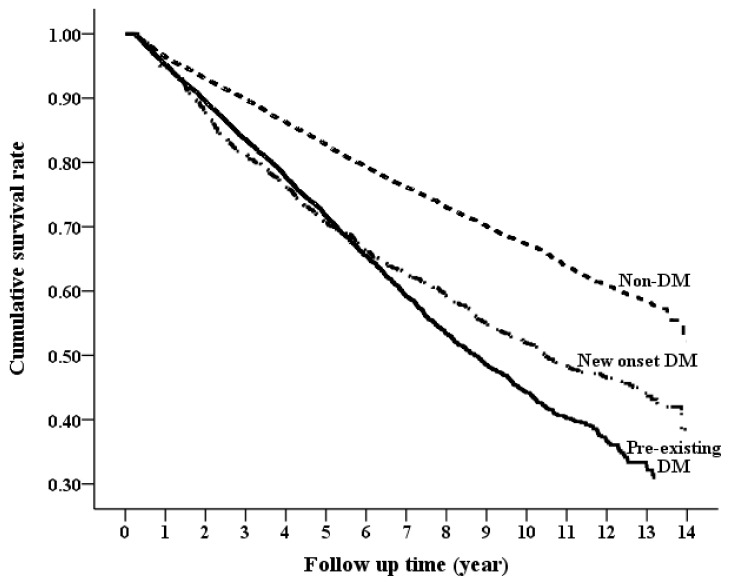
Cumulative overall survival curves between pre-existing DM, non-DM, and incident DM groups.

During the study period of 14 years, 2219 (21.43%) patients in the non-DM group died compared with 1895 (27.14%) in the pre-existing group and 303 (27.47%) in the non-DM group (*p* < 0.001). The cumulative survival curves for patients in the three DM groups using the Simon and Makuch method are shown in Figure 3. The incidence rate of death was 69.91 per 1000 person-years for the pre-existing DM group, 65.45 per 1000 person-years for the incident DM group, and 39.07 per 1000 person-years for the non-DM group, see Table 2. Pre-existing DM was associated with a 123% higher risk of death (HR 2.23; 95% CI 2.14–2.33), whereas incident DM was associated with a 148% higher risk of death (HR 2.48; 95% CI 2.37–2.60) when compared with the non-DM group. There was no significant difference in the risk of all-cause mortality between the incident DM and pre-existing DM groups (HR 0.99; 95% CI 0.95–1.04; Supplementary Table S2).

At the end of the study, 267 (2.58%), 364 (5.21%), and 44 (3.99%) patients had died of CVD in the non-DM, pre-existing DM, and incident DM groups, respectively (*p* < 0.001). A total of 714 (6.89%), 600 (8.59%), and 113 (10.24%) patients died of an infection in the non-DM, pre-existing DM, and incident DM groups, respectively (*p* < 0.001). The cumulative CVD and infection survival curves are shown in Supplementary Figures S1 and S2, respectively. Overall, both the pre-existing DM group and the incident DM group had a higher risk of CVD and infectious deaths compared with the non-DM group, as shown in Table 2.

### 3.4. Long-Term Risk of Composite Outcome (ESRD or Death) by DM Status

The incidence rate of the composite outcome was 143.66 per 1000 person-years for the pre-existing DM group, 95.4 per 1000 person-years for the incident DM group, and 63.34 per 1000 person-years for the non-DM group, see Table 2. Pre-existing DM was associated with a 129% higher risk of a composite outcome (HR 2.29; 95% CI 2.21–2.36), whereas incident DM was associated with a 77% higher risk of a composite outcome (HR 2.48; 95% CI 1.70–1.84) when compared with the non-DM group. However, when compared with the incident DM group, pre-existing DM was associated with a 35% higher risk of a composite outcome (HR 1.35; 95% CI 1.3–1.41; Supplementary Table S2).

### 3.5. Significant Risk Factors for Incident DM

A proportional Cox regression analysis was run to determine the risk factors for the development of incident DM in non-pre-existing DM patients (*n* = 11,459). As shown in Table 3, factors that positively contributed to incident DM included old age, low monthly income, the presence of hypertension, hyperlipidemia, and ischemic heart disease, whereas the use of pentoxifylline was associated with a reduced risk of incident DM.

## 4. Discussion

To the best of our knowledge, the present study was the first to use Taiwanese nationwide population-based data with proper longitudinal follow-up to investigate the differences in renal outcomes and patient survival among CKD patients who were stratified by DM status (non-DM, pre-existing, and incident DM). The key findings of the present study were as follows: (i) The presence of DM (pre-existing or incident) was associated with a higher risk of ESRD, mortality, and composite outcome (ESRD or mortality); (ii) the incident DM group had a mortality risk of all-cause death that was comparable with the pre-existing DM group; (iii) the pre-existing DM group had a higher risk of ESRD and composite outcome compared with the incident DM group.

DN, which is defined as CKD caused by DM, accounts for about 40% of all ESRD patients. In diabetic patients, CKD can be due to DN, non-diabetic renal disease (NDRD), or a combination of the two. A recent meta-analysis of 48 studies on the renal biopsy results of DM patients described a high prevalence of non-diabetic pathologies [20]. There has been an increase in research focusing on NDRD in patients with DM, as NDRD is being increasingly recognized as having distinct outcomes from DN. It has always been hypothesized that patients with NDRD had better outcomes due to a lower burden of CVD. However, in previous studies, it is not clear whether this increased survival is different between patients with DM contributing to primary kidney disease and those with DM as a comorbidity.

Previous studies evaluating the effect of DM status on the clinical prognosis of predialysis CKD patients are scarce. A community-based cohort study of elderly subjects showed that subjects with DM had a greater decline in eGFR than those without DM [21]. Recently, a Japanese study of 2484 CKD patients by Iwai et al. compared the renal outcomes among non-DM, DN, and NDRD groups [22]. They reported that DN patients had a higher risk of developing ESRD than NDRD and non-DM patients; they attributed the different outcomes between the NDRD and DN groups to the divergence in primary disease. Another study was conducted by Tan et al. that compared the renal and patient survival in 263 patients with type 2 DM who were categorized as DN, NDRD, or mixed type based on their renal biopsies [23]. Overall, patient survival was worse in the DN group compared with the NDRD and mixed groups, while the renal survival was better in the NDRD group compared with the DN and mixed groups. However, the results of the current study could not be compared with theirs, as renal biopsy results were not available in the present study. Tien et al. investigated the impact of DM status on dialysis patients using the same approach as the current study; that is, pre-existing DM, new-onset DM, and non-DM [24]. In multivariate analyses, they reported that pre-existing DM had the highest mortality risk followed by new-onset DM when compared with non-DM; however, they did not consider the competing risk of death and immortal time bias. The discrepancies between Tien’s results concerning mortality and the present study might be explained by differences in the study design, the statistical methods used, adjusted covariates, and the heterogeneity of the study populations.

Being old was a significant risk factor for incident DM in the current study CKD cohort. Age has been previously designated as a significant parameter for the diabetic risk score in the general population aged between 35 and 64 years [25]. The aging process contributes to DM inception through β cell dysfunction and increased insulin resistance [26]. Socioeconomic status was inversely associated with the risk of developing type 2 DM [27]. Factors contributing to the inverse relationship between income and incident DM may include psychological stress, lack of socioeconomic privileges, unemployment, unhealthy habits, financial hardship, and living in an at-risk neighborhood for those with lower incomes. The current study also demonstrated that ischemic heart disease, hyperlipidemia, and hypertension are independent risk factors for incident DM. Dyslipidemia and vascular atherosclerosis cause vascular inflammation and endothelial dysfunction, both of which contribute to an increased risk of type 2 DM [28]. Elevated levels of inflammatory markers, such as CRP or interleukin-6 have predicted the development of DM in previous studies [29]. A prospective study showed that endothelial dysfunction could independently predict DM [30]. One Italian study, which enrolled 8291 patients, reported an increased annual rate of incident DM for those with acute myocardial infarction [31]. Conen et al. conducted a prospective study to examine the association between hypertension and incident DM; they found that hypertension led to a higher risk of DM after adjusting for many components of metabolic syndrome [32]. Conversely, pentoxifylline, a nonselective phosphodiesterase inhibitor, was associated with a lower risk of DM. The exact underlying mechanism for this is unknown; however, it may be explained by the inhibitory effect of pentoxifylline on inflammation.

It is worth highlighting that the present study had a large sample size and utilized medical claims data that is verified through strict NHI reimbursement regulations. The DM status of the study population was classified as pre-existing DM, incident DM, or non-DM and the authors believe that this kind of classification is more reflective of clinical diagnoses in the real world. However, some limitations to the current study should be addressed. First, some established risk factors for type 2 DM include obesity, family history, unhealthy habits and diet pattern, a sedentary lifestyle, sleep disruption, and smoking. Many laboratory parameters which are important determinants for our outcome events consist of albuminuria, estimated glomerular filtration rate, and glycated hemoglobin. These factors were not adjusted for because the registry data did not contain information on them. Second, the time at risk started from the date of a CKD diagnosis, but it may take several years for CKD patients to develop DM from a non-DM status and patients must survive long enough to develop DM before death. To combat this, cause-specific time-dependent Cox models were used to mitigate the immortal time bias. Third, one may criticize the discrepancies in the distribution of demographic data and comorbidities across the three DM groups and claim they accounted for the different outcomes between them. Theoretically, pre-existing DM patients should have a worse outcome because they have had diabetes for a longer period with more severe multi-organ complications. However, IPW-integrated Cox models were used to overcome the problems of an unbalanced baseline covariate distribution. Fourth, the pathogenic mechanisms of type 1 and type 2 DM are different, so the impact of DM status on clinical outcomes could vary between type 1 and type 2 DM. Due to the nature of the registry data, we are unable to resolve this issue.

In conclusion, in a large cohort of patients from the nationwide registry data, it was found that the risk of death in CKD patients with incident DM was similar to CKD patients with pre-existing DM, higher than CKD patients with non-DM, and the risk of ESRD was higher than that of the non-DM group. These independent relationships were consistent after adjusting for the traditional prognostic factors, including cardiovascular comorbidities and medications. Establishing a diabetic risk score for pre-dialysis CKD patients may encourage a patient who gets a higher score to have their blood sugar concentrations checked more frequently. DM and CKD are both important public health concerns and undiagnosed DM is associated with a higher risk of mortality and CVD. Therefore, the incorporation of blood glucose testing into the routine laboratory tests for the integrated CKD care program could help recognize asymptomatic incident DM patients earlier. Closer surveillance with special attention to CKD patients at high risk for incident DM is necessary to improve clinical outcomes and enable treatment of potentially modifiable risk factors by implementing a healthy lifestyle.

## Figures and Tables

**Table 1:** Baseline characteristics and clinical outcomes of study patients by diabetic status.

	CKD Cohort				Maximum Standardization Difference between Groups	
	Non-DM	Pre-Existing DM	Incident DM	*p*-Value	Before IPW ^a^ (%)	After IPW ^a^ (%)
Sample size	10356	6982	1103	--	--	--
Age, years	65.45 ± 15.72	67.03 ± 12.02	65.21 ± 12.89	<0.001	0.128	0.037
Gender, Male	6254 (60.39%)	3834 (54.91%)	641 (58.11%)	<0.001	0.111	0.039
Monthly income, US dollars	471.28 ± 471.09	401.91 ± 386.44	409.58 ± 433.35	<0.001	0.158	0.090
Geographic location						
Northern	4465 (43.12%)	2973 (42.58%)	481 (43.61%)	0.705	--	--
Middle	2006 (19.37%)	1251 (17.92%)	214 (19.4%)	0.049	0.038	0.043
Southern	3599 (34.75%)	2540 (36.38%)	386 (35%)	0.086	0.034	0.051
Eastern	286 (2.76%)	218 (3.12%)	22 (1.99%)	0.079	0.068	0.044
Comorbidities within 1 year before the index date						
Hypertension	6362 (61.43%)	5509 (78.9%)	751 (68.09%)	<0.001	0.376	0.058
Hyperlipidemia	2465 (23.8%)	3137 (44.93%)	334 (30.28%)	<0.001	0.452	0.050
Ischemic heart disease	1973 (19.05%)	1799 (25.77%)	279 (25.29%)	<0.001	0.162	0.016
Congestive heart failure	1148 (11.09%)	1159 (16.6%)	137 (12.42%)	<0.001	0.163	0.019
Stroke	1273 (12.29%)	1264 (18.1%)	139 (12.6%)	<0.001	0.165	0.084
Rheumatoid disease	284 (2.74%)	101 (1.45%)	20 (1.81%)	<0.001	0.088	0.044
Cancer	831 (8.02%)	479 (6.86%)	59 (5.35%)	<0.001	0.102	0.041
COPD	1195 (11.54%)	655 (9.38%)	157 (14.23%)	<0.001	0.156	0.022
Charlson comorbidity index	2.16 ± 1.85	2.53 ± 1.89	2.07 ± 1.76	<0.001	0.246	0.076
Long-term medication use						
Anti-hypertensive drugs						
ACEI/ARB	3460 (33.41%)	4577 (65.55%)	376 (34.09%)	<0.001	0.645	0.057
beta-blocker	3109 (30.02%)	3127 (44.79%)	363 (32.91%)	<0.001	0.308	0.041
Diuretics	2366 (22.85%)	3095 (44.33%)	264 (23.93%)	<0.001	0.464	0.044
Statin	1570 (15.16%)	3343 (47.88%)	181 (16.41%)	<0.001	0.732	0.060
NSAIDs	1565 (15.11%)	1240 (17.76%)	148 (13.42%)	<0.001	0.118	0.018
Pentoxifylline	525 (5.07%)	756 (10.83%)	37 (3.35%)	<0.001	0.290	0.090
Outpatient visit within 1 year before the index date	29.16 ± 20.32	33.22 ± 20.94	31.28 ± 21.56	<0.001	0.196	0.055

Abbreviations: COPD, chronic obstructive pulmonary disease; ACEI, angiotensin-converting enzyme inhibitor; ARB, angiotensin II receptor blocker; NSAID, Non-Steroidal Anti-Inflammatory Drug. ^a^ Inverse probability of group-weighted (IPW) was estimated by the propensity score from generalized boosted regression. If a standardized difference of less than 10% is reached after IPW, the covariates are balanced.

**Table 2:** Risks for composite endpoint (ESRD or mortality), ESRD and mortality among patients with CKD by DM status.

Outcome	Non-DM		Pre-Existing DM		Incident DM		Time-Dependent Cox Model ^†^			
	Event	IR (95% CI)	Event	IR (95% CI)	Event	IR (95% CI)	Pre-Existing DM vs. Non-DM aHR (95% CI)	*p*-Value	Incident DM vs. Non-DM aHR (95% CI)	*p*-Value
Composite Endpoint	3294	63.34 (61.18−65.51)	3075	143.66 (138.58−148.74)	307	95.4 (84.72−106.07)	2.29 (2.21−2.36)	<0.0001	1.77 (1.70−1.84)	<0.0001
ESRD	1735	33.36 (31.79−34.93)	2168	101.28 (97.02−105.55)	111	34.49 (28.07−40.91)	2.54 (2.43−2.65)	<0.0001	1.12 (1.06−1.19)	0.0002
All-cause mortality	2219	39.07 (37.45–40.7)	1895	69.91 (66.76–73.06)	303	65.45 (58.08–72.82)	2.23 (2.14−2.33)	<0.0001	2.48 (2.37−2.60)	<0.0001
Cardiovascular death	267	4.70 (4.14−5.27)	364	13.43 (12.05−14.81)	44	9.50 (6.70−12.31)	3.00 (2.68−3.35)	<0.0001	2.68 (2.37−3.04)	<0.0001
Infection-related death	714	12.57 (11.65–13.49)	600	22.14 (20.36–23.91)	113	24.41 (19.91–28.91)	2.33 (2.16−2.52)	<0.0001	2.97 (2.74−3.21)	<0.0001

Abbreviation: CI = confidence interval; aHR = adjusted hazard ratio; IR = incidence rate (per 1000 person-years); ^†^ aHR was calculated from IPW-standardized time-dependent cause-specific Cox model, where the inverse probability of group-weighted (IPW) was estimated by the propensity of group from generalized boosted regression.

**Table 3:** The significant variables for new-onset DM after diagnosis of CKD in non–pre-existing DM patients (*n* = 11459).

Variables	Crude HR (95% CI)	*p*-Value	Adjusted HR ^†^ (95% CI)	*p*-Value
Age at diagnosis of CKD (years)	1.01 (1.01–1.02)	<0.0001	1.01 (1–1.01)	0.022
Gender, Male	0.949 (0.84–1.07)	0.387		
Monthly income	0.893 (0.85–0.94)	<0.001	0.93 (0.88–0.98)	0.003
Geographic location				
Northern	1			
Central	0.976 (0.83–1.15)	0.764		
Southern	1.042 (0.91–1.19)	0.548		
Eastern	1 (0.65–1.53)	0.999		
Comorbidities within 1 year before the index date				
Hypertension	1.532 (1.35–1.74)	<0.001	1.35 (1.18–1.55)	<0.0001
Hyperlipidemia	1.432 (1.26–1.63)	<0.001	1.36 (1.2–1.55)	<0.0001
Ischemic heart disease	1.425 (1.24–1.63)	<0.001	1.19 (1.03–1.37)	0.016
Congestive heart failure	1.4 (1.17–1.67)	<0.001		
Stroke	1.207 (1.01–1.44)	0.038		
Rheumatoid disease	0.684 (0.44–1.06)	0.092		
Cancer	0.904 (0.7–1.18)	0.453		
COPD	1.276 (1.08–1.51)	0.005		
Charlson comorbidity index	1.069 (1.03–1.11)	<0.001		
Long-term medication use				
ACEI/ARB	1.285 (1.13–1.46)	<0.001		
beta-blocker	1.328 (1.17–1.51)	<0.001		
Diuretics	1.338 (1.16–1.54)	<0.001		
Statin	1.337 (1.14–1.57)	<0.001		
NSAIDs	1.099 (0.92–1.31)	0.285		
Pentoxifylline	0.815 (0.59–1.13)	0.222	0.7 (0.51–0.98)	0.037
Outpatient visit within 1 year before the index date (per 1 visit)	1.006 (1–1.01)	<0.001		

^†^ adjusted HR was calculated from a cause-specific Cox model with a backward elimination procedure and variables with a *p* value < 0.3 in a univariate model were included in a multivariate model. Abbreviations: COPD, chronic obstructive pulmonary disease; ACEI, angiotensin-converting enzyme inhibitor; ARB, angiotensin II receptor blocker; NSAID, Non-Steroidal Anti-Inflammatory Drug.

## Supplementary Materials

The following are available online at http://www.mdpi.com/2077-0383/7/12/550/s1, Figure S1: Cumulative survival curves free of cardiovascular death between pre-existing DM, non-DM, and incident DM groups; Figure S2: Cumulative survival curves free of infectious death between pre-existing DM, non-DM, and incident DM groups; Table S1: ICD-9-CM codes used to identify chronic kidney disease, comorbidities and the cause of death; Table S2: Risks for Composite endpoint (ESRD or mortality), ESRD and mortality among patients with CKD by DM status.
