# Supplementary material for: Effect of New-Onset Diabetes Mellitus on Renal Outcomes and Mortality in Patients with Chronic Kidney Disease
Source: J Clin Med. 2018 Dec 14;7(12):550. doi: 10.3390/jcm7120550 (PMC6306867; doi:10.3390/jcm7120550)
Supplement: Supplementary file 1 [file jcm-07-00550-s001.pdf]

Supplementary Table 1. ICD-9-CM codes used to identify chronic kidney disease, comorbidities and the cause of death.

Diseases	Corresponding ICD-9-CM codes
Chronic kidney disease	585
Diabetes mellitus	250.x
Co-morbid diseases	
Hypertension	401.x–405.x
Hyperlipidemia	272.x
Ischemic heart disease	410.x–414.x
Congestive heart failure	428.x
COPD	491.x, 492.x, 496.x
Rheumatoid disease	446.5, 710.0-710.4, 714.0-714.2, 714.8, 725.x
Cancer	140-165, 170-172, 174-195, 200-208
Stroke	430.x–438.x
**Causes of Death**	
Cardiovascular death	390.x – 398.x, 410.x – 414.x, 4151, 41511, 41519, 420.x, 422.x – 429.x, 431.x, 433.x – 436.x, 518.4, 785.51
Infection-related death	001.x – 139.x, 320.x, 321.x, 326.x, 421.x, 460.x – 466.x, 480.x – 487.x, 510.x, 513.x, 551.x, 567.x, 590.x, 599.x, 680.x – 686.x, 711.x, 730.x
Other causes	Diagnostic codes other than cardiovascular and infection-related death

Abbreviation: COPD, chronic obstructive pulmonary disease; ICD-9-CM, International Classification of Disease, 9th Revision, Clinical Modification.

Supplementary Table 2: Risks for Composite endpoint (ESRD or mortality), ESRD and mortality among patients with CKD by DM status

Outcome	Time-dependent Cox's Model†			
	Pre-existing DM vs. Incident DM aHR (95% CI)	P-value	Non-DM vs. Incident DM aHR (95% CI)	P-value
Composite Endpoint	1.35(1.3-1.41)	<.0001	0.60(0.57-0.62)	<.0001
ESRD	2.30(2.17-2.44)	<.0001	0.89(0.84-0.95)	0.0003
All-cause mortality	0.99(0.95-1.04)	0.7802	0.44(0.42-0.46)	<.0001
Cardiovascular death	1.21(1.09-1.36)	0.0006	0.40(0.35-0.45)	<.0001
Infection-related death	0.88(0.82-0.95)	0.001	0.37(0.34-0.40)	<.0001

Abbreviation: CI = confidence interval; aHR = adjusted hazard ratio; IR =incidence rate (per 1000 person-years);

†aHR was calculated from IPW-standardized time-dependent cause-specific Cox model, where the inverse probability of group-weighted (IPW) was estimated by the propensity of group from generalized boosted regression.

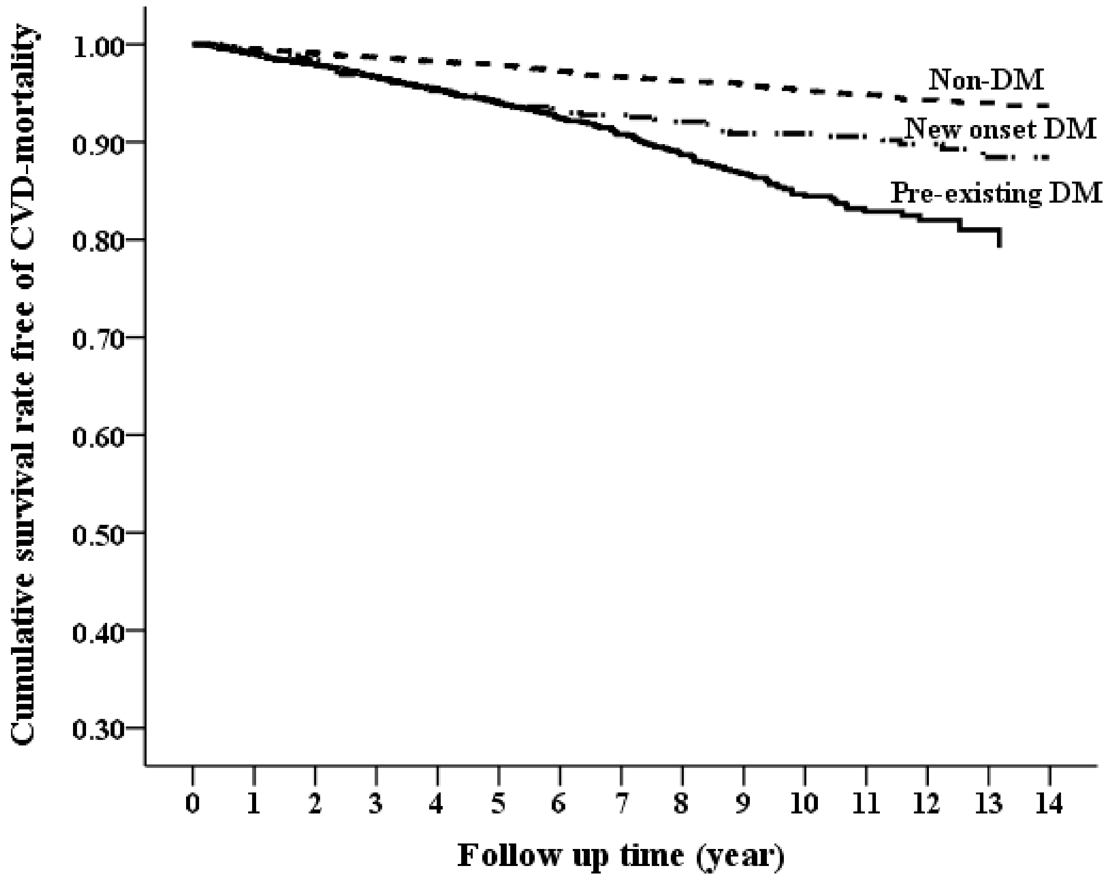

Supplementary Figure 1. Cumulative survival curves free of cardiovascular death between pre-existing DM, non-DM and incident DM groups.

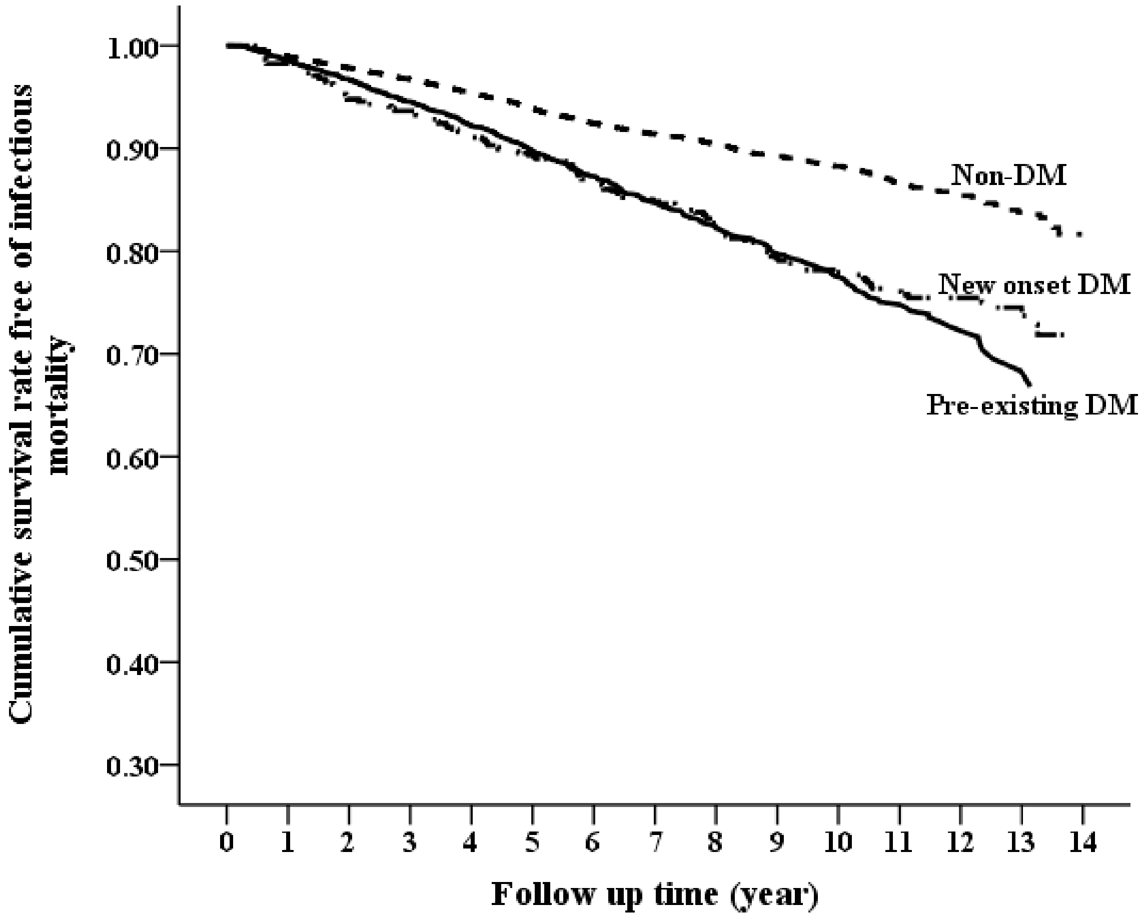

Supplementary Figure 2. Cumulative survival curves free of infectious death between pre-existing DM, non-DM and incident DM groups.
